# Imaging Pitfall in the Pediatric Knee: Irregular Epiphyseal Ossification at the Femoral Condyle

**DOI:** 10.5334/jbsr.2673

**Published:** 2021-12-10

**Authors:** Frederiek Laloo, Marcela De La Hoz Polo, Saira Haque

**Affiliations:** 1 Ghent University Hospital, BE; 2 King’s College Hospital, London, GB

**Keywords:** Magnetic Resonance Imaging, Pediatrics, Osteogenesis, Femur, Epiphyses, Osteochondritis dissecans

## Abstract

**Teaching point:** Age-related variability in endochondral ossification of the femoral condyles in children is a normal variant of skeletal maturation and should not be misdiagnosed as osteochondritis dissecans or any other epiphyseal abnormality.

## Case

A 9-year-old boy was referred to the accident and emergency department. He had sustained an acute left knee injury with severe pain and restriction of movement. No previous medical history was known.

**Figure 1:**
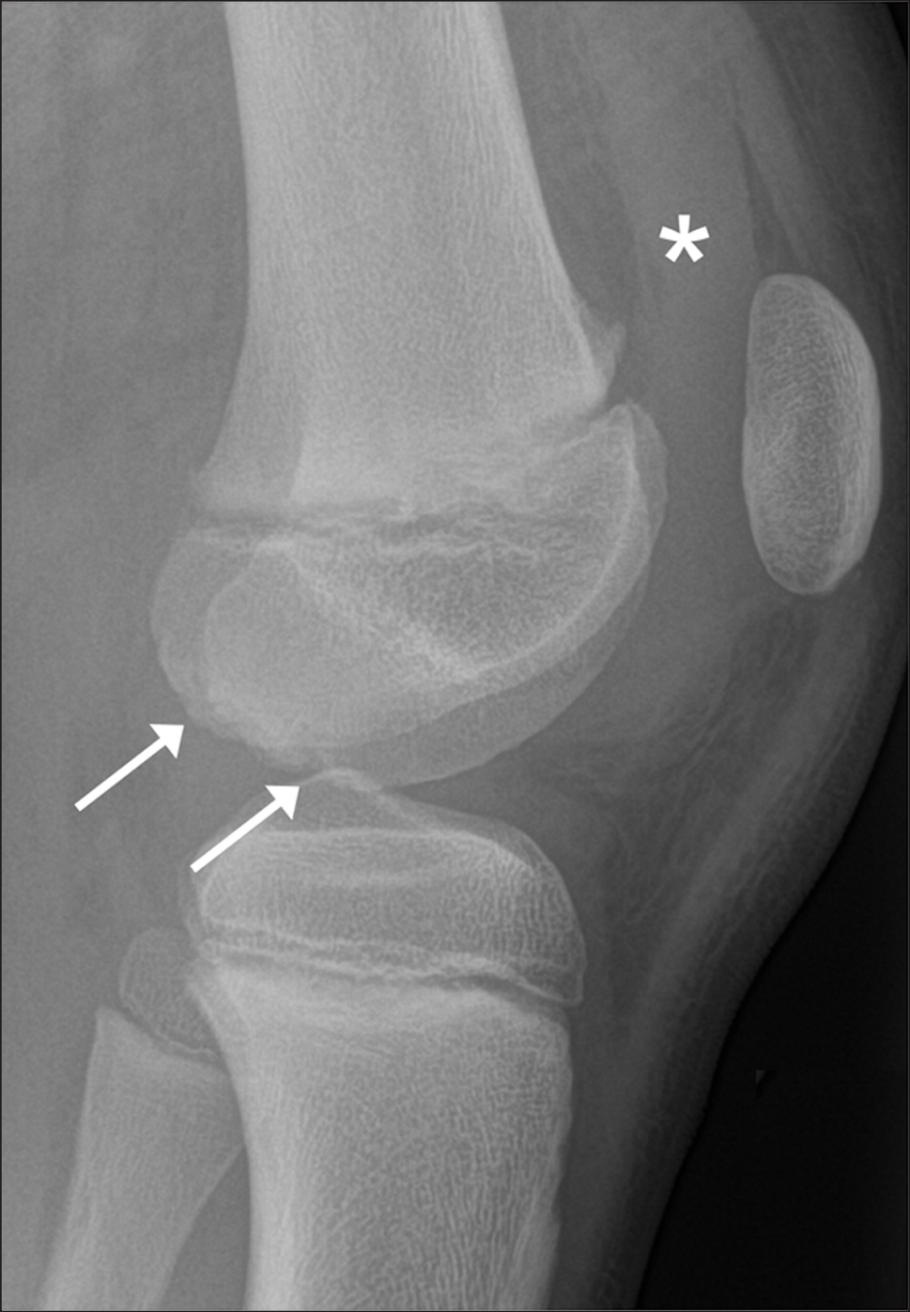


Lateral radiograph of the right knee demonstrated a large joint effusion (***Figure 1**, asterisk*) without any evident fracture. Irregular epiphyseal outline at the posterior aspect of both femoral condyles was also noted (***Figure 1**, arrows*).

**Figure 2:**
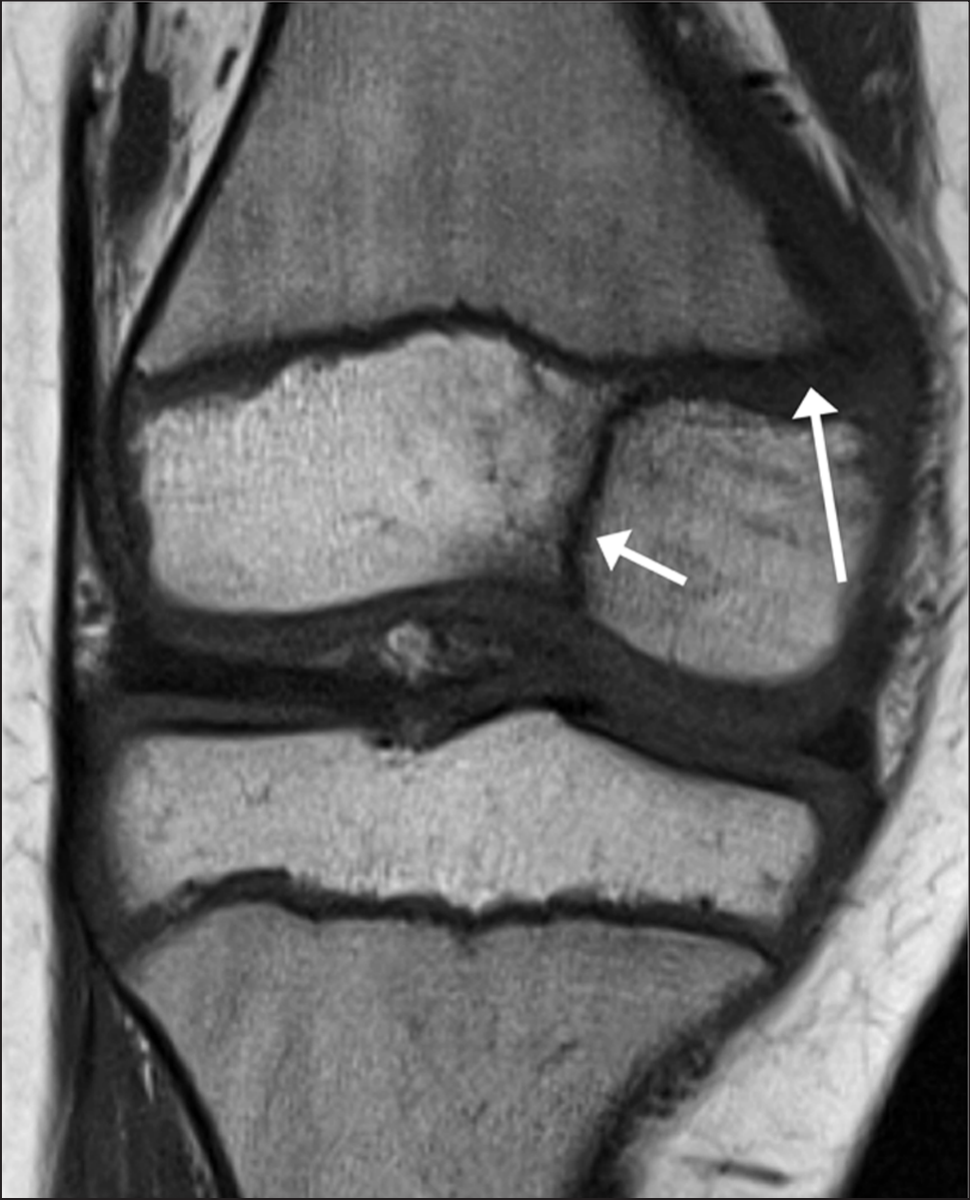


MRI of the right knee – proton density (PD) and fat suppressed proton density (FS-PD) – were obtained to evaluate for radiographically occult fractures and meniscal or ligamentous injury. MRI evidenced a Salter-Harris type III fracture (***Figure 2**, PD-sequence, short arrow*) with widening of the growth plate (***Figure 2**, PD-sequence, long arrow*).

**Figure 3:**
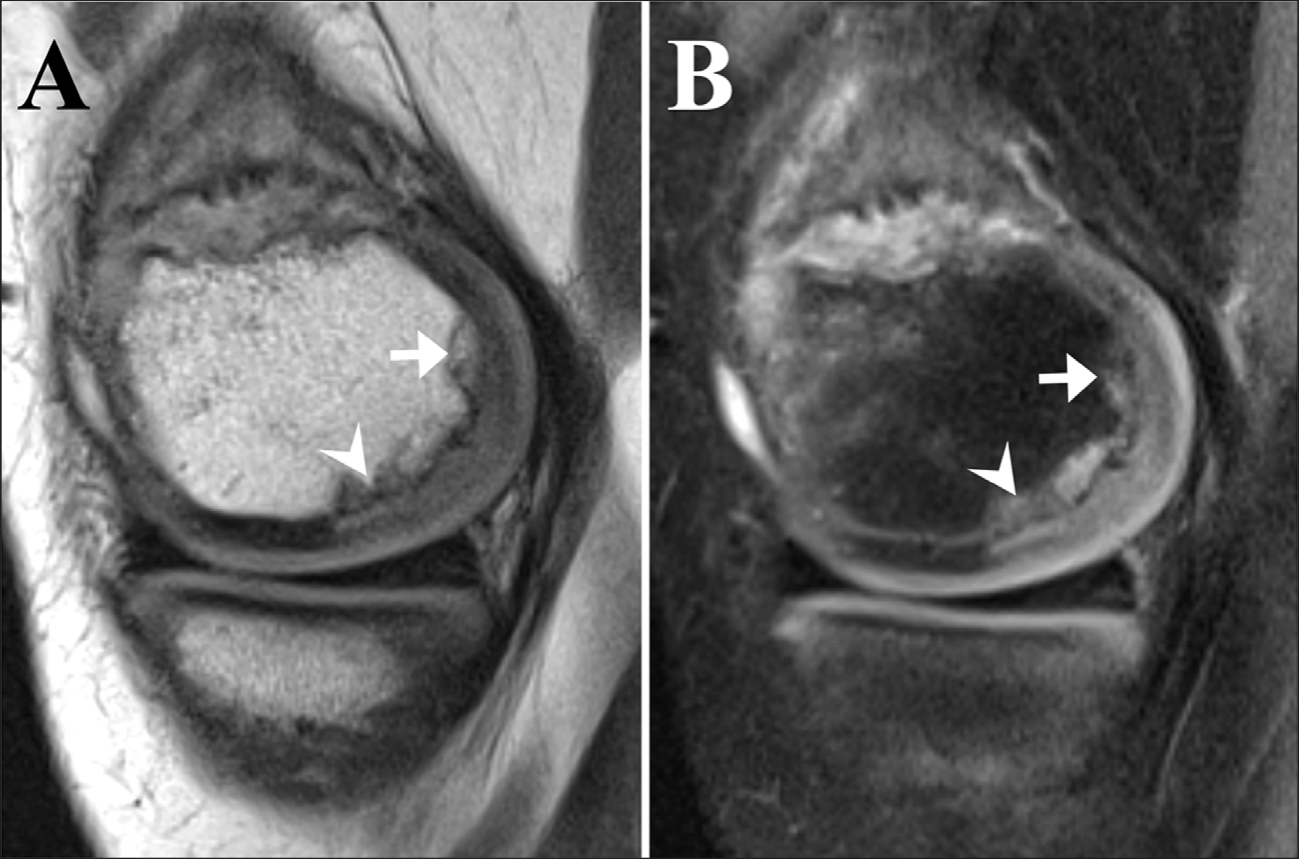


Additionally, ill-defined defects in the posterior third of both femoral condyles were present (***Figure 3a** and **3b**, PD and FS-PD sequences*) representing incomplete epiphyseal bone formation – a normal variant. The defects at the more cranial aspect of the lateral femoral condyle showed a more advanced stage of ossification (***Figure 3a** and **3b**, PD and FS-PD sequences, arrow*), while the defects at the more caudal aspect of the femoral condyle showed a more spiculated and early ossification (***Figure 3a** and **3b**, PD and FS-PD sequences, arrowhead*). Note the intact overlying cartilage and the absence of bone marrow oedema.

## Comment

Variability in endochondral ossification of articular cartilage is a normal variant of skeletal maturation at secondary ossification centres and is more often seen in boys (mean age of 8 years old) than in girls (mean age of 6 years old) [1]. Eventually, the lobulated foci of ossification will merge, and the bone will obtain homogenous signal intensity and a regular outline.

Irregular epiphyseal ossification of the femur is often bilateral and typically located at the posterior aspect of the femoral condyle. This variant can also be seen in other bones, such as the trochlea of the elbow and the tarsal navicular. When unilateral, osteochondritis dissecans or a fracture are the main pitfalls for misdiagnosis – lack of adjacent bone marrow oedema and intact overlying cartilage are key differentiating features on MRI. It is important to correctly recognise this normal morphologic variability and distinguish it from pathological epiphyseal disease, which may lead to incorrect treatment and unnecessary follow-up investigations.
